# Bedaquiline and Delamanid Combination Treatment of 5 Patients with Pulmonary Extensively Drug-Resistant Tuberculosis

**DOI:** 10.3201/eid2310.170834

**Published:** 2017-10

**Authors:** Andrey Maryandyshev, Emanuele Pontali, Simon Tiberi, Onno Akkerman, Shashank Ganatra, Tsetan Dorji Sadutshang, Jan-Willem Alffenaar, Rohit Amale, Jai Mullerpattan, Sonam Topgyal, Zarir Farokh Udwadia, Rosella Centis, Lia D’Ambrosio, Giovanni Sotgiu, Giovanni Battista Migliori

**Affiliations:** Northern State Medical University, Arkhangelsk, Russia (A. Maryandyshev); Galliera Hospital, Genoa, Italy (E. Pontali);; Royal London Hospital of Barts Health National Health Service Trust, London, UK (S. Tiberi);; Queen Mary University of London, London (S. Tiberi);; University of Groningen, Haren, the Netherlands (O. Akkerman, J.-W. Alffenaar);; P.D. Hinduja National Hospital and Medical Research Centre, Mumbai, India (S. Ganatra, R. Amale, J. Mullerpattan, Z.F. Udwadia);; Delek Hospital, Dharamshala, India (T.D. Sadutshang, S. Topgyal);; Maugeri Care and Research Institute, Tradate, Italy (R. Centis, L. D’Ambrosio, G.B. Migliori);; Public Health Consulting Group, Lugano, Switzerland (L. D’Ambrosio);; University of Sassari, Sassari, Italy (G. Sotgiu)

**Keywords:** MDR TB, XDR TB, bedaquiline, delamanid, combination treatment, effectiveness, safety, tolerability, Europe, the Netherlands, India, Russia, Italy, United Kingdom, Switzerland, pulmonary, bacteria, *Mycobacterium tuberculosis*, multidrug-resistant tuberculosis, tuberculosis, mycobacteria, antimicrobial resistance, respiratory infections, multidrug resistance, TB

## Abstract

We report the experiences of 5 patients taking bedaquiline with delamanid in combination: 1 patient was cured; 3 culture converted, with 2 continuing and 1 changing therapy; and 1 died from respiratory insufficiency. For 2 patients, QT-interval prolongation but no arrhythmias occurred. Use of this therapy is justified for patients with limited options.

According to the World Health Organization (WHO), 480,000 multidrug-resistant (MDR) tuberculosis (TB) and 100,000 rifampin-resistant TB cases, and 250,000 deaths attributable to these 2 conditions, occurred globally in 2015 (*1*). About 10% of the bacteria isolates from MDR TB cases met the criteria for extensively drug-resistant (XDR) TB (resistance to any fluoroquinolone and >1 second-line injectable drugs) (*1**,**2*).

MDR TB and XDR TB treatments are of long duration, expensive, and complicated by a high rate of adverse events, making determining an effective drug regimen often difficult, considering that a minimum of 4 active drugs are required according to WHO recommendations (*1**–**4*). In this regard, bedaquiline (*5**,**6*) and delamanid (*7*) might be crucial for designing effective treatment regimens.

Although these drugs are increasingly used in combination in complicated cases (*8**–**11*), public health officials are concerned that the co-administration of bedaquiline and delamanid could increase the occurrence of adverse events, particularly for QT prolongation, which might occur more often when these drugs are combined with other TB drugs that prolong the QT interval (i.e., fluoroquinolones and clofazimine). Only 2 reports describe the co-administration of these drugs (*8**–**11*). As of July 2017, the WHO does not recommend their combined use, given the lack of evidence regarding their safety (*4*).

MDR TB reference centers belonging to the International Bedaquiline Study Group (25 centers located in 15 countries in Africa, Asia, Western and Eastern Europe, Oceania, and South America working within the framework of the European Respiratory Society, the Asociación Latinoamericana de Tórax, and the Brazilian Society collaborative projects) (*12*) performed a large study investigating safety, tolerability, and effectiveness of bedaquiline-containing regimens for MDR and XDR TB patients treated through and not through national TB programs. However, no information on co-administration of bedaquiline and delamanid was included. We conducted a retrospective and observational subanalysis of patients from the International Bedaquiline Study Group study who were undergoing treatment with bedaquiline and delamanid.

## The Study

 We consecutively enrolled patients ≥15 years of age from the International Bedaquiline Study Group study who underwent treatment during January 1, 2008–August 30, 2016, on the basis of their exposure to both bedaquiline and delamanid during the intensive and/or continuation phase of the study. Bedaquiline was administered at the recommended dosage of 400 mg/d for 14 days and then 200 mg 3×/wk with delamanid at 200 mg/d. We obtained ethics approval for this retrospective research from the coordinating center and each clinical center that enrolled the patients as required by law; patients and attending physicians signed consent forms agreeing to participate.

The following were considered adverse events: an absolute QT interval corrected with Fridericia’s formula (QTcF) prolongation of >500 ms; a QTcF increase of >60 ms over the baseline reading; cardiac arrest; ventricular tachycardia or atrial fibrillation; syncope; and events suggestive of arrhythmia, dizziness, seizures, and palpitations. To assess severity, we used the Common Terminology Criteria for Adverse Events version 4.0 (https://evs.nci.nih.gov/ftp1/CTCAE/CTCAE_4.03_2010-06-14_QuickReference_8.5x11.pdf).

Of the 428 patients with culture-confirmed MDR TB who were treated with bedaquiline, 5 received combined treatment with delamanid. Considering the long half-life of bedaquiline (>5 months), 2 additional patients could have also been considered to have combined treatment; they were treated with delamanid shortly after bedaquiline (*5**–**6*). On April 28, 2017, we obtained information on the patients’ last follow-up from the physicians managing their care, and this information was updated in the study database.

Bedaquiline and delamanid were given concurrently to 5 patients with pulmonary XDR TB who lived in Russia (2), India (2), or the Netherlands (1) (Table 1). Four were women and 1 was a man; patients were 17–43 years of age. All were HIV negative, and 2 were recreational drug users. All had previously been treated with TB drugs (range 1–8 treatments) for >30 days; 4 patients had drug treatment failures, and 1 had a relapse. Chest radiographs indicated that 3 patients had extensive bilateral cavities, 1 had bilateral lesions (without cavities), and 1 had monolateral cavitary lung disease. All patients were sputum-smear and culture positive for mycobacteria and had been potentially infectious for a mean of 65 weeks.

**Table 1:** Demographics and clinical history of patients with pulmonary extensively drug-resistant TB treated with bedaquiline and delamanid*

Pt no.	Country of birth/illness	Age, y/ sex	Risk factor	No. treatments >30 d, case category	Weeks ss+ and c+ before Bdq + Dlm treatment	Weight at baseline (last recorded), kg	MDR TB treatment duration, mo	Length of hospital stay, d	Previous TB drug regimen	Drug resistance before Bdq + Dlm (at end of study)
1	India/India	20/F	None	1, failure	200	34 (40)	50	NA	Cm, Mfx, Eto, PAS, Cfz, Lzd, Cs, Rfb, Bdq	S, H, R, E, Z, Fq, PAS, Km, Rfb (Lzd, Eto)
2	UK/the Netherlands	31/F	Recreational drug user	8, failure	4	54 (68)	21	567	H, R, Z, E, Amk, Cm, Cfz, Pto, PAS, Cs, Mpm, Amx/Clv, Clr	S, H, R, E, Z, Fq, Eto, Amk, Lzd
3	Russia/ Russia	43/M	Recreational drug user	1, failure	62	54 (76)	36	887	Cm, Z, Mfx, Trd, Pto, PAS	H, R, E, Fq, Km
4	Azerbaijan/Russia	17/F	None	1, failure	20	53 (51)	16	256	Z, Cm, Lfx, Pto, Cs, PAS	H, R, E, Z, Km, Amk, Cm, Fq
5	Tibet/India	39/F	None	2, relapse	52	65 (60)	18	1,140	H, R, Z, E, Hd H, Mfx, Km, PAS, Lzd, Pto	R, Km, Amk, Cm, Fq, Eto, PAS, Lzd, Hd H, Hd Mfx

*All patients were sputum smear and culture positive. Amk, amikacin; Amx/Clv, amoxicillin/Clavulanate; Bdq, bedaquiline; c+, culture positive; Cfz, clofazimine; Clr, clarithromycin; Cm, capreomycin; Cs, cycloserine; Dlm, delamanid; E, ethambutol; Eto, ethionamide; Fq, fluoroquinolone; H, isoniazid; Hd, high dose; Km, kanamycin; Lfx, levofloxacin; Lzd, linezolid; MDR TB, multidrug-resistant tuberculosis; Mfx, moxifloxacin; Mpm, meropenem; NA, not available; PAS, para-aminosalicylic acid; Pt, patient; Pto, prothionamide; R, rifampin; Rfb, rifabutin; S, streptomycin; ss+, sputum smear positive; TB, tuberculosis; Trd, terizidone; Z, pyrazinamide.

The resistance patterns of the isolated *Mycobacterium tuberculosis* strains were extensive, ranging from 5 to 10 drugs (Table 1). Salvage regimens were designed for each patient on the basis of their unique resistance patterns, which lead to their treatments including bedaquiline and delamanid (Table 2). All regimens included another QT-prolonging drug in addition to bedaquiline and delamanid: moxifloxacin (patients 1 and 3) or clofazimine (patients 2, 4, and 5). Patient exposure to bedaquiline was 155–427 days, for a total duration of TB treatment of 16–46 months. The total duration of hospital admission was 256–1,140 days. 

**Table 2:** Summary of patients treated with bedaquiline and delamanid, including data on the anti-TB regimen administered, bacteriological conversion, treatment outcomes, and QT interval monitoring*

Pt no.	Last TB drug regimen administered	Sputum smear/culture conversion, d (treatment outcome)	Dlm/Bdq exposure, d	QT before treatment, ms	QT average, ms (±SD)	QT max, ms (wk)
1	Cm, Mfx, Eto, Cs, PAS, Cfz, Mpm, Lfx, Amx/Clv, Lzd, Bdq, Dlm	NA/NA (failure; 4 mo after completing Bdq + Dlm treatment course, patient died because of respiratory insufficiency)	168/168	410	426 (±17.6)	450 (9)
2	Hd H, Cfz, Cs, E, Lzd, Dlm, Bdq; as of April 28, 2017, receving: Hd H, Cs, Cfz, cotrimoxazole	60/60 (continued treatment)	168/168	400	406 (±33.6)	462 (24)
3	Cm, Mfx, Bdq, Dlm, Lzd, Imp, Amx/Clv	435/104 (cured)	180/180	340	363 (±25.8)	400 (35 and 51)
4	Bdq, Dlm, Lzd, Cfz	30/30 (continued treatment)	155/155	394	462 (±39.8)	509 (5 and 9)
5	Dlm, Bdq, Cfz, Trd, Mpm, Amx/Clv	18/28 (continued treatment)	427/427	449	504 (±6.3)	520 (16)†

*Amk, amikacin; Amx/Clv, amoxicillin/clavulanate; Bdq, bedaquiline; Cfz, clofazimine; Cm, capreomycin; Cs, cycloserine; Dlm, delamanid; E, ethambutol; Eto, ethionamide; H, isoniazid; Hd, high dose; Imp, imipenem; Lfx, levofloxacin; Lzd, linezolid; max, maximum; Mfx, moxifloxacin; Mpm, meropenem; NA, not achieved; PAS, para-aminosalicylic acid; Pto, prothionamide; Pt, patient; QT, measure of the time between the start of the Q wave and the end of the T wave in the heart's electrical cycle; SD, standard deviation; TB, tuberculosis; Trd, terizidone. †At different time points, intermittent episodes of asymptomatic QTc prolongation occurred.

As of April 28, 2017, patient 3 had been declared cured; patients 2, 4, and 5 were continuing therapy, although patient 2 was receiving a different drug regimen. Patient 1 had received 4 months of salvage therapy, but treatment failed, and she died from respiratory insufficiency. Patient 2 switched therapies because bedaquiline and delamanid had been already administered for a fixed period of 168 days as recommended by WHO. With the exception of patient 1, who remained sputum-smear and culture positive, the other 4 patients’ sputum smears converted to negative after 18–435 days, and cultures converted after 28–218 days.

As recommended, all patients underwent QTcF-interval monitoring at baseline, at 2 weeks, and then monthly (*4*), even though no patient had a history of heart problems or electrocardiogram abnormalities. A QTc interval >500 ms is considered a risk factor for fatal arrhythmia; when this sign is found in patients, clinicians should either stop treatment with >1 QTc-prolonging drugs and start verapimil or watch and closely monitor. The baseline QTcF intervals were <500 (range 340–449) ms for all patients. Patients 1, 2, and 3 did not report adverse events for bedaquiline or delamanid, and their QTcF intervals remained below the threshold. Patient 5’s QTcF interval reached 520 ms at week 16, which required a dose adjustment and the introduction of verapamil (*9**–**11*). Patient 5’s treatment continued without further problems; she continued improving clinically, with improved chest radiograph findings and continuously negative sputum smears and cultures. Patient 4 had a QTcF interval of 509 ms twice. Each time the treating physician practiced closer clinical observation with more frequent electrocardiogram monitoring, and her QTcF interval normalized spontaneously without changes in treatment.

## Conclusions

We report that of 5 patients receiving bedaquiline and delamanid in combination 2 had potentially life-threatening QTcF prolongation. The clinical centers took the necessary precautions and acted promptly to manage the problem, and no arrhythmias occurred (*9*–*11*). When patients received bedaquiline, delamanid, and another QTc-prolonging agent, clinically significant cardiac events and permanent discontinuation of bedaquiline and delamanid did not occur. For patient 1, additional resistance to ethionamide and linezolid was detected in a drug susceptibility test in the final phase. This treatment failure highlights that great care is needed when deciding drug regimens; the resistance threshold of both repurposed and new drugs still needs to be determined. Although these data are preliminary and more work is needed, the findings from this cohort suggest that providing bedaquiline and delamanid in combination as part of therapy against XDR TB is justified when clinical options are limited. Two ongoing randomized controlled trials (ClinicalTrials.gov nos. NCT02583048 and NCT02754765) have experimental arms containing these drugs in combination, so additional datasets will be available in the future.
